# Emotion Recognition Based on Dynamic Energy Features Using a Bi-LSTM Network

**DOI:** 10.3389/fncom.2021.741086

**Published:** 2022-02-21

**Authors:** Meili Zhu, Qingqing Wang, Jianglin Luo

**Affiliations:** Modern Animation Technology Engineering Research Center of Jilin Higher Learning Institutions, Jilin Animation Institute, Changchun, China

**Keywords:** EEG, emotion recognition, dynamic energy feature, Bi-LSTM, energy sequence

## Abstract

Among electroencephalogram (EEG) signal emotion recognition methods based on deep learning, most methods have difficulty in using a high-quality model due to the low resolution and the small sample size of EEG images. To solve this problem, this study proposes a deep network model based on dynamic energy features. In this method, first, to reduce the noise superposition caused by feature analysis and extraction, the concept of an energy sequence is proposed. Second, to obtain the feature set reflecting the time persistence and multicomponent complexity of EEG signals, the construction method of the dynamic energy feature set is given. Finally, to make the network model suitable for small datasets, we used fully connected layers and bidirectional long short-term memory (Bi-LSTM) networks. To verify the effectiveness of the proposed method, we used leave one subject out (LOSO) and 10-fold cross validation (CV) strategies to carry out experiments on the SEED and DEAP datasets. The experimental results show that the accuracy of the proposed method can reach 89.42% (SEED) and 77.34% (DEAP).

## Introduction

Emotion mainly refers to a kind of inner emotional response produced by the psychological needs of people. It is a series of subjective psychological experiences formed by integrating the feelings, perceptions, and behaviors of people (Li, 2017). In the daily life and work of people, emotion is everywhere. In medical care, different nursing measures can be provided according to the emotions of patients to improve the quality of care. When treating psychological and physical diseases, an emotional state can be used as an auxiliary diagnostic basis. In the human-computer interaction, by identifying emotional states, the interaction is more friendly and natural. Therefore, the qualitative recognition and classification of emotional states of people have a high research value.

Psychological and emotional states can be transmitted in a variety of signal forms, such as *via* facial expressions, voice intonation, text, body posture, and other directly observable emotional signals. An emotional state of a person can also be learned through physiological signals, such as electrical brain signals, skin temperature, and breathing signals. Compared with observable emotions, electroencephalogram (EEG) signals are obtained by directly reading brain activity. EEG signals are more real and difficult to disguise. Therefore, the results obtained by using EEG signals to classify emotions are more authentic and reliable (Ullsperger et al., 2014; Naseer and Hong, 2015; Chen et al., 2020). As a result, this article mainly discusses studying emotion recognition methods based on EEG signals.

In EEG emotion recognition research, the first type of method is based on traditional machine learning technology. This kind of method first expands the emotional feature extraction from the EEG signal and then expands the corresponding emotional pattern learning and classification of the feature data. Emotional feature extraction of EEG signals can be divided into four categories, including time-domain features, frequency-domain features, time-frequency features, and EEG signal non-linear dynamic features. For example, Atkinson et al. proposed an EEG emotion recognition method based on feature selection and a kernel classifier (Atkinson and Campos, 2016). In this study, the statistical characteristics of the EEG signal, frequency band power of different frequencies, Hjorth parameter, and fractal dimension are used. The statistical features include median, SD, and kurtosis coefficient. The frequency bands of EEG signals use θ (4–8 Hz), low α (8–10 Hz), α (8–12 Hz), β (12–30 Hz), and γ (30–45 Hz). Then, a feature selection method based on mutual information and a kernel classifier are used to optimize the performance of the emotion classification task. Mohammadi et al. decomposed the EEG signal using a wavelet transform, while the entropy feature and the energy feature were extracted from each sub-band signal. Then, the support vector machine (SVM) and *k*-nearest neighbor (KNN) pattern classifiers were used for training and testing (Mohammadi et al., 2017). The results showed that the accuracy of arousal was 86.75%, and the accuracy of valence was 84.05%. Fernández-Varela et al. evaluated the emotion recognition performance of six machine learning models in detail as follows: Fisher’s linear discriminant, SVM, artificial neural network, classification tree, KNN, and naive Bayes (Isaac et al., 2017). Li et al. (2018) studied the effectiveness and performance of 9 time-frequency domain features and 9 non-linear dynamic features through the combination of different feature selection and SVMs. Abhishek Tiwari et al. proposed motif series and graph-theoretical features, before analyzing three feature selection methods [ANOVA-based feature ranking and selection, minimum redundancy maximum relevance (mRMR) feature selection, and recursive feature elimination (RFE)] and three fusion strategies (feature fusion, score-level fusion, and output-associated fusion). They finally concluded that motif series features are better than spectral features and that feature-level fusion strategies can improve recognition accuracy. Score-level fusion can improve arousal prediction (Tiwari and Falk, 2019). Fu Yang et al. constructed a multidimensional high-dimensional feature set by extracting eight linear features, such as the Hjorth parameter, power spectral density, and SD, and two non-linear features, such as wavelet entropy and sample entropy. Then, the ST-SBSSVM method was proposed by combining the significance test, sequential backward selection, and the SVM (Yang et al., 2019). The accuracy of the ST-SBSSVM was 72% (DEAP) and 98% (SEED).

The second type of method is based on deep learning technology. Deep learning has powerful feature extraction and classification capabilities. The main deep learning models used in the field of EEG signal classification include convolutional neural networks (CNNs), recurrent neural networks (RNNs), and generative adversarial networks (GANs). For example, Wang and Shang (2013) used a deep belief network (DBN) to extract features from the original physiological data and constructed three classifiers to estimate the arousal, valence, and liking of emotions. Yang et al. (2018) and Banee et al. (2019) put 32 electrodes into a 9 × 9 matrix according to the position of the international 10–20 system. The former used a time sliding window to divide the continuous EEG signal and calculated the average value of the corresponding electrode in each window. The latter directly inputs the original signal value into the matrix. The final input of the CNN network is a two-dimensional matrix containing spatial location information. Li et al. (2017) used a sparse wavelet transform to transform the EEG signals of each channel into a two-dimensional spectrum and then input them into a CNN-RNN hybrid network. Hwang et al. (2020) generated an egg image with topological information based on differential entropy (DE) features and then used a deep network structure combining a CNN and an LSTM. Shawky et al. (2018) converted EEG signals into a three-dimensional continuous frame input depth network, which compared EEG signals with images while ignoring the spatial position information of electrodes in EEG signals. Ant topic et al. proposed two feature mapping methods, called topography (TOPO-FM) and holography (HOLO-FM). Then, the deep learning technique was employed for feature learning from TOPO-FM and HOLO-FM, and finally, SVM was used to classify emotions (Topic and Russo, 2021). In the above research, the transformation of the EEG feature form imitated the image learning mode of deep networks, including a CNN, an RNN, and a DBN as much as possible. In addition, there are studies on EEG signal analysis by using brain function networks, such as energy landscape (Krzemiński et al., 2020; Klepl et al., 2021), which provides a new idea for the study of EEG signals.

Electroencephalogram signals are non-stationary, non-linear, and complex physiological signals with multicomponent mechanisms (Golyandina et al., 2001). In the machine learning method, the time domain, frequency domain, and time-frequency domain characteristics ignore the fact that the EEG signal is a non-linear signal, and non-linear dynamic features need to extract sufficient structural information of EEG components. In addition, the traditional machine learning method extracts features from multiple channels of EEG signals and then connects them to a single feature vector without considering the temporal dynamics. In the method based on deep learning, it is difficult to train a high-quality model due to the small number of samples and the low resolution of EEG images. In addition, the feature analysis and extraction of EEG signals are easily affected by noise, which impacts the effect of the deep learning model. Therefore, this study discusses combining the features extracted by manual calculation with a neural network to solve two problems. The first problem is simply and effectively representing multichannel EEG signals before establishing the emotion recognition model. The second problem is constructing effective features to learn temporal dynamics. We regarded brain emotion processing as an overall coordinated physiological response. To save artificial features while retaining the original information as much as possible, we proposed emotion recognition based on dynamic energy features using a Bi-LSTM network, and the main contributions are as follows:

(1) The concept of energy sequence is proposed. Dynamic feature extraction based on the energy sequence can effectively avoid the noise superposition effect caused by the feature extraction of a single channel sequence.
(2) The feature set is constructed by the combination of mutual information and principal component analysis (PCA). The feature set can reflect the time persistence of the EEG signal and the complexity of the multicomponent structure. The network model adopts fully connected layers and long short-term memory (LSTM) networks, which are suitable for small datasets.

## Materials and Methods

### Experimental Data

The datasets used in this study are SEED and DEAP. SEED (Duan et al., 2013; Zheng and Lu, 2015) is a collection of EEG datasets provided by the BCMI laboratory. The dataset recorded 62 channels of EEG data at a sampling frequency of 1,000 Hz (200 Hz resampling) and collected 15 individuals (7 men and 8 women), as well as three kinds of emotional EEG data (positive, negative, and neutral). The size of the dataset is 3 × 15 × 15 × 62 × 4,800 (3 experiments, 15 videos/trials, 62 channels, and (200 Hz × 240 s) data). The DEAP (database for emotion analysis using physical signals) was collected by Koelstra et al. (2012) from Queen Mary University in London, the University of Twente in the Netherlands, the University of Geneva in Switzerland, and the Federal Institute of Technology in Switzerland. The dataset recorded 40 channels of physiological signals at a sampling frequency of 512 Hz (128 Hz resampling) and collected the data of four emotions (valence, arousal, dominion, and liking) of 32 people (16 men and 16 women). The size of the dataset was 32 × 40 × 40 × 8,064 (40 videos/trials, 40 channels, and 128 Hz × 63 s).

### Data Preprocessing

**FIGURE 1:**
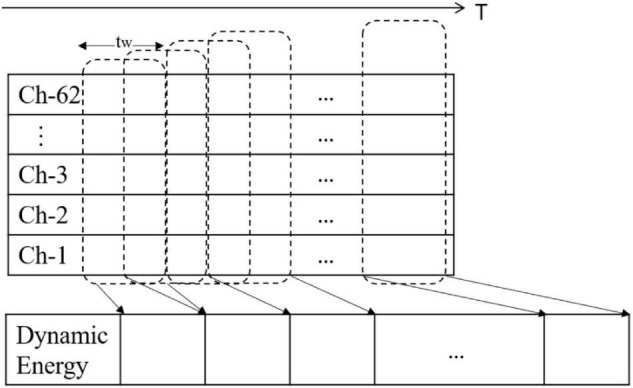
Data preprocessing.

Single-channel EEG signals can achieve strong results in attention state detection, sleep state detection, and identity recognition. Taran and Bajaj (2019) also obtained good results in emotion recognition through a single-channel signal. Inspired by this, this study combines the spatial distribution of the whole head electrode to preserve the integrity of cerebral cortex information. The data preprocessing is shown in Figure 1.

First, the spatial information of different channels is fused, and the concept of a dynamic energy sequence is proposed as follows:


$$ETs\left(t\right)=\frac{1}{N}\sum_{i=1}^{N}s\left(t,i\right)$$

where *N* represents the channels and *s*(*t*, *i*) is the value of time *t* in the *i*-th channel. The energy sequence projects the time sequence in multiple channels onto an equal-length energy sequence.

Assuming that the time signal of the *i*-th channel is*s*(*t*, *i*)and the noise is *n*(*t*, *i*), the noise and the signal space are independent of each other and distributed independently. Then, the time signal affected by the noise is as follows:


$$s^{\prime }\left(t,i\right)=s\left(t,i\right)+n\left(t,i\right)$$

The average value of the energy sequence is as follows:


$$E\left(ETs^{\prime }\left(t\right)\right)=E\left(\frac{1}{N}\sum_{i=1}^{N}\left[s\left(t,i\right)+n\left(t,i\right)\right]\right)=E\left(\frac{1}{N}\sum_{i=1}^{N}s\left(t,i\right)+\frac{1}{N}\sum_{i=1}^{N}n\left(t,i\right)\right)=\bar{s}\left(t\right)+\bar{n}\left(t\right)$$

where $\bar{s}\left(t\right)$represents the average value of the sequence and $\bar{n}\left(t\right)$ represents the average value of the noise.

The variance of the energy series is as follows:


$$\sigma_{ETs^{\prime }\left(t\right)}^{2}=E\left({\left[ETs^{\prime }\left(t\right)-E\left(ETs^{\prime }\left(t\right)\right)\right]}^{2}\right)$$

$$=E\left({\left\{\frac{1}{N}\sum_{i=1}^{N}\left[s\left(t,i\right)+n\left(t,i\right)\right]-\left[\bar{s}\left(t\right)+\bar{n}\left(t\right)\right]\right\}}^{2}\right)$$

$$=E\left({\left\{\left[\frac{1}{N}\sum_{i=1}^{N}s\left(t,i\right)-\bar{s}\left(t\right)\right]+\left[\frac{1}{N}\sum_{i=1}^{N}n\left(t,i\right)-\bar{n}\left(t\right)\right]\right\}}^{2}\right)$$

$$=\sigma_{ETs\left(t\right)}^{2}+\frac{1}{N}\sigma_{n\left(t\right)}^{2}$$

$$=\frac{1}{N}\sigma_{s\left(t\right)}^{2}+\frac{1}{N}\sigma_{n\left(t\right)}^{2}$$

Equations (2) and (3) show that the mean value of the energy sequence after noise influence is the same as that of the single-channel sequence, and the variance is 1/*N* of the single-channel sequence. In the traditional EEG sequence recognition process, each channel is extracted separately, and then it is recognized or trained. During the recognition process, the noise will be superimposed, and the noise interference is much greater than that of single-channel sequence noise. Therefore, the method of projecting the sequence of multiple channels to the energy sequence can effectively smooth the noise, avoid the noise superimposition effect caused by extracting the features of the single-channel sequence separately, and improve the robustness.

During the procedure of EEG signal processing, even if preprocessing methods are used, such as artifact removal, downsampling, filtering, and component decomposition, the EEG signal noise still remains. Therefore, this study ignores the asymmetry of the brain emotion processing function, regards brain emotion processing as an overall coordinated physiological response, projects the time sequences of multiple channels to the energy time sequence, and saves the cost of artificial feature extraction while retaining the original information as much as possible. In our later experiments, considering the different activation degrees of different channels, the contribution to the template is also different. Therefore, we divided the brain lobe into 4 regions, which are the left anterior region, right anterior region, left posterior region, and right posterior region. According to the conclusion in the study (Li et al., 2018; Zheng et al., 2019), the area with high emotional activation response is set with a high coefficient. The specific region division and coefficient setting are shown in Table 1.

**TABLE 1:** Regional division and coefficient setting.

	SEED	DEAP	a_*i*_
1	FP1,AF3,F7,F5,F3,F1,FT7, FC5,FC3,FC5,T7,C5,C3,C1	EP1,AF3,F7,F3, FC5,FC1,T7,C3	$\sum_{i=1}^{M_{1}}a_{i}=0.4$
2	FP2,AP4,F2,F4,F6,F8 FC2, FC4,FC6,FC8,FPZ,FZ,FCZ,CZ	FP2,AF4,F4,F8, FC2,FC6,Fz,Cz	$\sum_{i=1}^{M_{2}}a_{i}=0.3$
3	TP7,CP5,CP3,CP1,P7,P5,P3,P1, PO7,PO5,PO3,CB1,O1,CPZ,PZ,POZ,OZ	CP5,CP1,P7,P3, PO3,01,Pz,Oz	$\sum_{i=1}^{M_{3}}a_{i}=0.2$.
4	CP2,CP4,CP6,TP8,P2,P4,P6,P8,PO4, PO6,PO8,CB2,O2,C2,C4,C6,C8	CP2,CP6,P4,P8, PO4,O2,C4,T8	$\sum_{i=1}^{M_{4}}a_{i}=0.1$.

Second, emotion is a continuous physiological and psychological process and state, and past emotional states may have an impact on the current emotions. To combine the time persistence of the emotional state and obtain the dynamic attributes of the emotional characteristics of EEG signals as much as possible, we introduced the time window variable *t*_*w*_ on the time axis. The moving time window method is used to intercept the sequence fragments from the time series. We calculated the energy sequence corresponding to the same time window.

For SEED, we intercepted the data from the 2nd min to the 3rd min, and for DEAP, we intercepted the data from the 4th to the 63rd min. The relevant data settings are shown in Table 2.

**TABLE 2:** Data parameter setting.

Array name	SEED	DEAP
Number of Channels	62	32
Length of time	60	60
Time window width	4s	4s
Time window move step	2s	2s
Number of time windows	29	29
Number of sample points in time window	800	512
Label type	2/3	2

### Dynamic Energy Feature Set

**FIGURE 2:**
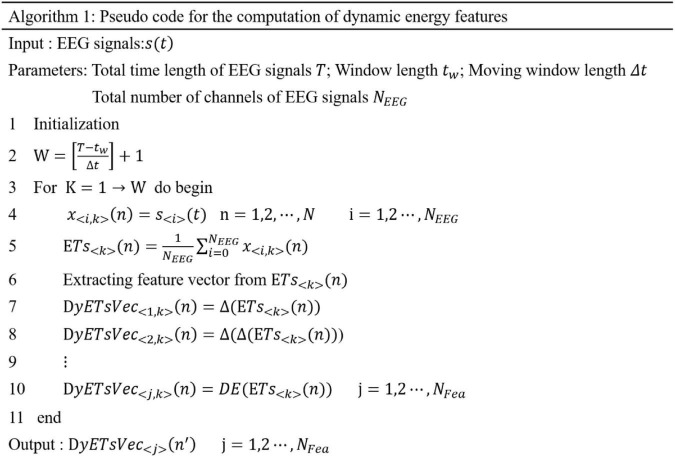
Pseudocode for computing dynamic energy feature set.

Based on the dynamic energy sequence, we extracted different types of features from each energy time sequence and then form the feature set according to the time sequence. The feature set formed by different types of features extracted from the dynamic energy sequence is a time-domain profile of multicomponent features, which can reflect the time-domain dynamic characteristics of EEG emotional signals and the complexity of the multicomponent structure. The pseudocode of the dynamic energy characteristic calculation is shown in Figure 2.

In this study, seven kinds of time-domain features, three kinds of frequency-domain features, and three kinds of dynamical features are extracted.

#### Time-Domain Features

The EEG signal is a discrete sequence obtained by sampling a continuous signal according to a certain sampling frequency through an EEG device. Suppose that *s*(*n*) represents the EEG signal value obtained by sampling a certain electrode, *n* = 1, 2, 3 … , *N*. *N* represents the total number of samples, μ_*s*_ represents the average value, and σ_*s*_ represents the SD.

(1) The average value is calculated as follows:

$$\mu_{s}=\frac{1}{N}\sum_{n=1}^{N-1}s\left(n\right)$$

(2) The SD is calculated as follows:

$$\sigma_{s}=\sqrt{\frac{1}{N}\sum_{n=1}^{N}{\left(s\left(n\right)-\mu_{s}\right)}^{2}}$$

(3) The first-order differential is calculated as follows:

$$\delta_{s}=\frac{1}{N-1}\sum_{n=1}^{N-1}\left|s\left(n+1\right)-s\left(n\right)\right|$$

The normalized first-order differential (Yao, 2015) is calculated as follows:


$$\bar{\delta_{s}}=\frac{\delta_{s}}{\sigma_{s}}$$

(4) The second-order differential is calculated as follows:

$$\gamma_{s}=\frac{1}{N-2}\sum_{n=1}^{N-2}\left|s\left(n+2\right)-s\left(n\right)\right|$$

The normalized second-order differential is calculated as follows:


$$\bar{\gamma_{s}}=\frac{\gamma_{s}}{\sigma_{s}}$$

(5) The instability index (Kroupi et al., 2011) is calculated as follows:

*s*(*n*) is divided into *M* segments, and the mean value of each segment is calculated. The SD of these *M* mean values is defined as NSI.

(6) The energy is calculated as follows:

$$E_{s}=\sum_{n=1}^{N}{\left|s\left(n\right)\right|}^{2}$$

(7) The fractal dimension (Ansari-Asl et al., 2007) is calculated as follows:

We can express *s*(*n*) and *n* = 1, 2, 3 … , *N* as $\left\{s\left(m\right),s\left(m+k\right),\ldots ,s\left(m+\left\lfloor \frac{N-m}{k}\right\rfloor \cdot k\right)\right\}$, where *m* = 1, 2, 3 … , *k* is the start time and *k* is the time interval, which can be seen as follows:


$$H_{m}\left(k\right)=\frac{N-1}{\left[\frac{N-m}{k}\right]k^{2}}\sum_{i=1}^{\left[\frac{N-m}{k}\right]}\left|s\left(m+ik\right)-s\left(m+\left(i-1\right)k\right)\right|FD_{s}=-\frac{\log\bar{H\left(k\right)}}{\log k}$$

where $\bar{H\left(k\right)}$ denotes the mean of *H*_*m*_(*k*).

#### Frequency Domain Features

(1) Hjorth parameters (Bo, 1970):

First, the EEG signal is transformed by a discrete Fourier transform (DFT). Then, the Hjorth parameters are calculated in theta (4–7 Hz), alpha (8–13 Hz), beta (14–30 Hz), gamma (31–50 Hz), and the full band.

The DFT is calculated as follows:


$$S\left(k\right)=DFT\left[s\left(n\right)\right]=\sum_{n=0}^{N-1}s\left(n\right)W_{N}^{nk}=\sum_{n=0}^{N-1}s\left(n\right)e^{-j\left(\frac{2\pi }{N}\right)nk}$$

The activity is as follows:


$$A_{c}=\frac{1}{N}\sum_{k=0}^{N-1}{\left(S\left(k\right)-\bar{S\left(k\right)}\right)}^{2}$$

The mobility is as follows:


$$M_{o}=\sqrt{\frac{var\left(S^{\prime }\left(k\right)\right)}{var\left(S\left(k\right)\right)}}$$

The complexity is as follows:


$$C_{o}=\frac{M_{o}\left(S^{\prime }\left(k\right)\right)}{M_{o}\left(S\left(k\right)\right)}$$

(2) Power spectrum and power spectral density:

In this study, the autoregressive model (AR mode) is used to estimate the power spectrum of EEG signals, and the process is as follows:

*s*(*n*)is expressed by the difference equation as $s\left(n\right)=-\sum_{k=1}^{p}a_{k}s\left(n-k\right)+\omega \left(n\right)$,

where ω(*n*)represents the white noise sequence with zero mean and $\sigma_{\omega }^{2}$ variance. *k*represents the order of the AR model.

The transfer function of the AR model is as follows:


$$H\left(z\right)=\frac{1}{1+\sum_{k=1}^{p}a_{k}z^{-k}}$$

The power spectrum is as follows:


$$P_{s}\left(\omega \right)=\frac{\sigma_{\omega }^{2}}{{\left|A\left(e^{j\omega }\right)\right|}^{2}}=\frac{\sigma_{\omega }^{2}}{{\left|1+\sum_{k=1}^{p}a_{k}e^{-j\omega k}\right|}^{2}}$$

#### Dynamical Features

(1) Approximate entropy (Pincus, 1991):

Constructing the vector $X_{i}^{\left(m\right)}=\left(x_{i},x_{i+1},x_{i+2}\cdots x_{i+m-1}\right)$, 1 ≤ *i* ≤ N-m + 1, *N* represents the length of the time series, *m* represents the embedded dimension, then counts the number of vectors *C^m^*(*i*, *r*) similar to vector $X_{i}^{\left(m\right)}$, and *r* is the similarity tolerance. The method is as follows:


$$C^{m}\left(i,j,r\right)=\left\{ \begin{array}{c} 1\begin{array}{cc} , & d\left(X_{i}^{\left(m\right)},X_{j}^{\left(m\right)}\right)<r \end{array}\\ 0\begin{array}{cc} , & otherwise \end{array} \end{array}\right.$$

$$C^{m}\left(i,r\right)=\sum_{j=1}^{N-m}C^{m}\left(i,j,r\right)$$

Now the formula is defined as follows:


$$P_{i}^{m}\left(r\right)=\frac{1}{N-m+1}C^{m}\left(i,r\right),$$

$$\Phi^{m}\left(r\right)=\frac{1}{N-m+1}\sum_{i=1}^{N-m+1}\ln\left(P_{i}^{m}\left(i\right)\right)$$

We can repeat the above steps to calculate Φ^*m* + 1^(*r*); then, the approximate entropy is as follows:


$$ApEn=\Phi^{m}\left(r\right)-\Phi^{m+1}\left(r\right)$$

(2) Wavelet entropy (Sharma et al., 2015):

In this study, the DB4 wavelet transform is used to decompose the EEG signal in six levels, and then the Shannon entropy is extracted from the approximate component and the detail component.

(3) Differential entropy (Zheng and Lu, 2015):

$$DE=\int_{-\infty }^{+\infty }\frac{1}{\sqrt{2\pi \sigma_{i}^{2}}}e^{-\frac{{\left(x-\mu \right)}^{2}}{2\sigma_{i}^{2}}}\log\left(\frac{1}{\sqrt{2\pi \sigma_{i}^{2}}}e^{-\frac{{\left(x-\mu \right)}^{2}}{2\sigma_{i}^{2}}}\right)dx=\frac{1}{2}\log\left(2\pi e\sigma_{i}^{2}\right)$$

### Feature Dimension Reduction

Feature dimension reduction includes feature extraction and feature selection, which is very important for recognition and classification. The effective dimensionality reduction method can not only improve the speed of the algorithm but also filter out irrelevant attributes and reduce the interference of irrelevant information with classification to improve the accuracy of the classification and prevent overfitting.

The basic idea of feature dimensionality reduction is to score each feature vector according to some evaluation function, rank the feature vectors according to the score value from high to low, and take some feature vectors with a high score value as a feature set. In this study, we considered the effectiveness of a single feature label and the information redundancy between multiple features. We adopted the method of combining mutual information (Hossain et al., 2014) and PCA (Ian and Jorge, 2016) to reduce dimensionality. The process is as follows:

Suppose that the feature space of the sample dataset *X* is *R*^*m*×*n*^, each row of data *X*_*i*_ is (*x*_*i*1_, *x*_*i*2_, *x*_*i*3_, ⋯ *x*_*in*_), and *x*_*ij*_ represents the *j*-th feature value of the *i*-th sample.

Step 1: Calculate the proportion of the value of the *i*-th sample on the *j*-th feature to the overall value of the *j*-th feature as follows:


$$p\left(x_{i}\right)=\frac{x_{ij}}{\sum_{i=1}^{m}x_{ij}}$$

Step 2: Calculate the information entropy of the *i*-th sample as follows:


$$H\left(x_{i}\right)=-\sum_{i=1}^{n}p\left(x_{i}\right)\log p\left(x_{i}\right)$$

Step 3: Calculate the mutual information matrix as follows:


$$I\left(X,Y\right)=-\sum_{i=1}^{n}\sum_{j=1}^{m}p\left(x_{i},y_{j}\right)\log\frac{p\left(x_{i},y_{j}\right)}{p\left(x_{i}\right)p\left(y_{j}\right)}$$

$$\sum_{I_{xy}}=\left[\begin{array}{ccc} I_{11} & \cdots & I_{n1}\\ \vdots & \ddots & \vdots \\ I_{n1} & \cdots & I_{nn} \end{array}\right]$$

Step 4: The matrix decomposition is as follows:


$$\sum_{I_{xy}}=B^{\prime }\Lambda B$$

where Λ is the diagonal matrix composed of the eigenvalues (μ_1_, μ_2_, ⋯ μ_*n*_) of ∑_*I_xy_*_ and *B* is a matrix composed of eigenvectors (β_1_, β_2_, ⋯ , β_*n*_) corresponding to the eigenvalues.

Step 5: Calculate the contribution rate σ_*k*_ and the cumulative contribution rate δ_*k*_ as follows:


$$\sigma_{k}=\frac{\mu_{k}}{\sum_{k=1}^{n}\mu_{k}},\delta_{k}=\sum_{k=1}^{n}\sigma_{k}$$

Step 6: Select new features.

The eigenvectors (β_1_, β_2_, ⋯ , β_*l*_) corresponding to the first *k* eigenvalues with 85–95% contribution rates are selected as the principal component decision matrix. The new feature matrix is as follows:


$$Z=B_{l}X$$

### The Network Structure

**FIGURE 3:**
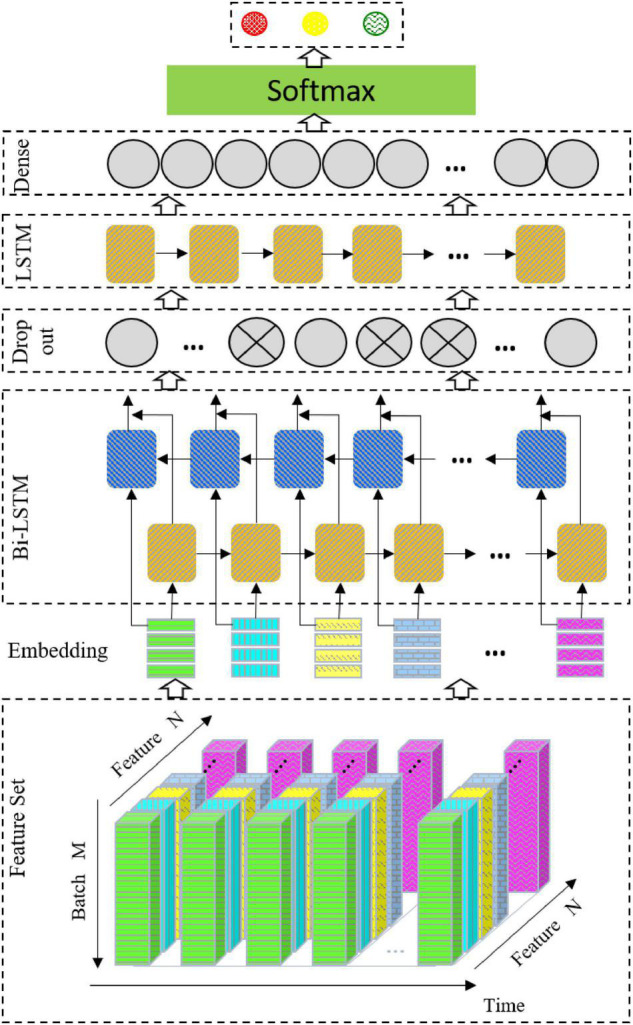
Schematic diagram of network structure.

The network structure used in this study is shown in Figure 3. The architecture consists of a Bi-LSTM network, a dropout layer, an LSTM network, and a full connection layer output.

#### Bidirectional Long Short-Term Memory

Bidirectional LSTM (Bi-LSTM) is based on LSTM (Hochreiter and Schmidhuber, 1997), which combines the information of the input sequence in both the forward and backward directions. In this study, the vector outputs by the two LSTM layers are connected and sent to the next layer. The Bi-LSTM is selected for the following reasons. First, mental emotion is a continuous physiological and psychological process and state. EEG signals are time-dependent. Therefore, emotion recognition of EEG signals may require the information of the whole input sequence, and the Bi-LSTM can better capture the context information in the sequence. Second, manual feature extraction is when the signal extracted from the EEG time series signals uses a sliding time window, which still belongs to a time series and can be modeled by the Bi-LSTM. Finally, for EEG signal recognition, the number of samples is small. Even when using data enhancement, it is difficult to train a good depth network model, and the Bi-LSTM is suitable for small datasets.

#### Dropout

To prevent overfitting, the dropout layer (Hinton et al., 2012) is linked after the Bi-LSTM layer. In each iteration, the neuron is randomly output to zero according to the specified proportion.

#### Long Short-Term Memory

To further extract features, the output data of the dropout layer is sent to the LSTM.

Parameter settings of the model are shown in Table 3.

**TABLE 3:** Model parameter setting.

Array name	SEED	DEAP
Input_size	17	19
Bi-LSTM	128	128
Drop out	0.3	0.3
LSTM	64	64
Dense 1	128	128
Dense 2	3/2	3
Optimizer	RMSprop	RMSprop
Learning rate	0.001	0.001
Epochs	46	55

## Results

To verify the feasibility and effectiveness of the proposed method, we performed four groups of experiments: comparison with traditional methods, feature type selection, individual emotion recognition, and comparison with other methods.

(1) Comparison with traditional methods:

The experiment is carried out by using the feature extraction method proposed in this study. The contrast method is the traditional method of extracting features from a single-channel signal. The methods are as follows: extracting features from signals in each channel, then using PCA for dimension reduction, and finally using SVM for recognition. To input the features into the SVM model, the feature vectors are composed of the mean value of the corresponding eigenvalues in the time window. In this way, the time-domain dynamics are considered to a certain extent. The relevant parameters of SVM are as follows: the type is C-SVC, the kernel function is RBF, and the setting method of penalty coefficient is Scan the parameter space 2^[–3:8]^ in step 1 to search for the best value.

**FIGURE 4:**
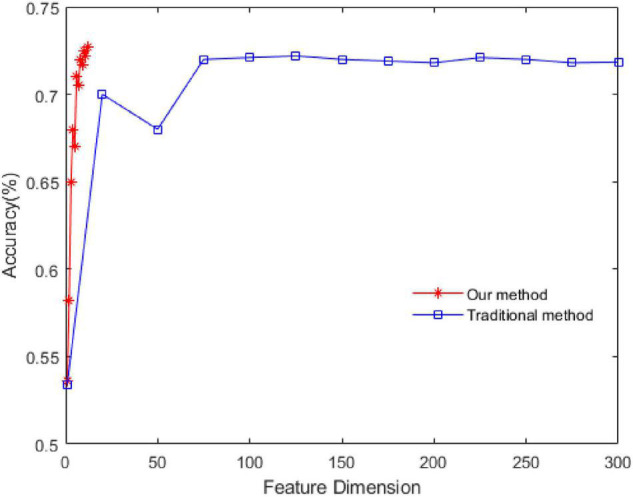
Comparison of recognition rates between proposed and traditional methods.

The experimental dataset is SEED, and positive and negative emotions are identified. The results are shown in Figure 4.

In this study, we first obtained the energy sequence of each channel and then extracted features from the energy sequence. Therefore, the feature dimension of the energy sequence is 1/62 of that of the single channel. As seen in Figure 4, the recognition rate of the proposed method is 72.5% and that of the traditional method is 72.2%, which shows that the proposed dynamic energy sequence can retain the characteristics of each channel of the EEG signal and effectively reduce the feature dimension.

**FIGURE 5:**
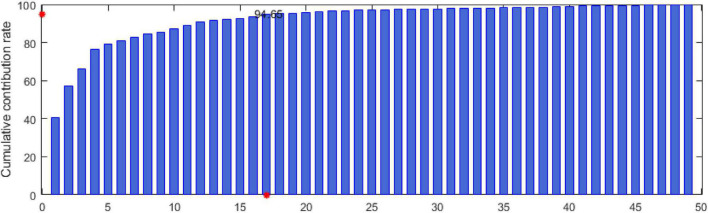
Cumulative contribution rate of principal components.

(2) Feature type selection:

In this study, the data of 6 subjects in the seed dataset are randomly selected. According to the feature extraction method in the “Dynamic energy feature set” section, 49 types of features are extracted. Then, the MIPCA method is used to analyze the feature set *F*^270×49^. The cumulative contribution rate of the principal components is shown in Figure 5. In Figure 5, the *x*-axis represents the principal component serial number, and the *y*-axis represents the cumulative contribution rate. The *y*-axis red mark indicates that the cumulative contribution rate has reached 94.65, and the *x*-axis red mark indicates that the number of principal components when the cumulative contribution rate is 94.65. In our experiment, the principal components whose cumulative contribution rate is before 95% are selected to determine the final feature set. Considering that different subjects may have different suitable features, we conducted three experiments at random and conducted statistical analysis on the results. The final features are shown in Table 4.

**TABLE 4:** Top features contributing to the prediction of emotion using the MIPCA method for SEED.

Feature type	SEED
Time domain features	1. The first-order differential, 2. The normalized first-order differential, 3. The second-order differential, 4. The normalized second-order differential, 5. Instability index, 6. Fractal dimension, 7. Hjorth-mobility, and 8. Hjorth-complexity
Frequency domain features	1. Hjorth-mobility (beta), 2. Hjorth-mobility (all), 3. Hjorth-complexity (beta), 4. Hjorth-complexity (all), 5. Maximum power spectrum (beta) frequency, and 6. Maximum power spectrum (all) frequency
Dynamical features	1. De-gamma, 2. De-all, and 3. Wavelet entropy (all)

It can be seen from Table 4 that among the selected features, there are 8 time-domain features with the largest number. This shows that the time-domain features have a strong resolution. In the frequency domain, the features in beta, gamma, and all bands are better than those in other bands. This is consistent with the conclusion in the study by Li et al. (2018). For the DEAP dataset, in addition to the features in Table 4, we added the wavelet entropy (gamma) and the power spectrum sum (all).

**FIGURE 6:**
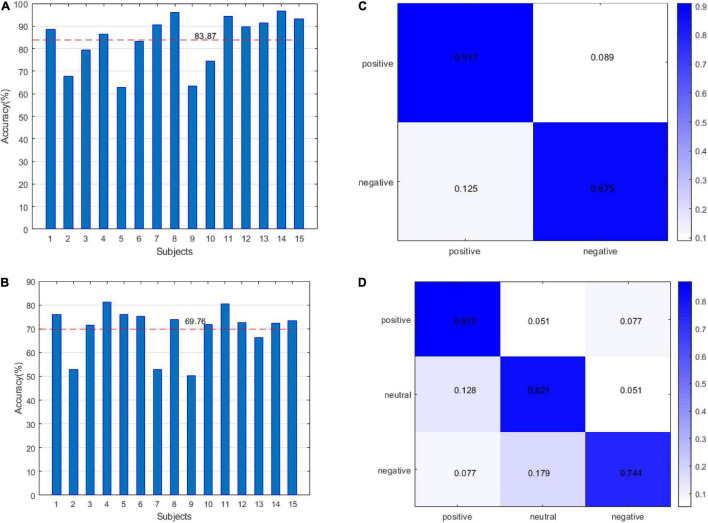
The results of the SEED datasets: **(A)** leave one subject out (LOSO) for the 2-classification. **(B)** LOSO for the 3-classification classes. **(C)** 10-fold CV for the 2-classification. **(D)** 10-fold CV for the 3-classification.

(3) Emotion recognition:

In this study, we adopted two verification strategies, namely, leave one subject out (LOSO) and 10-fold cross validation (CV). For SEED, we classified two-dimensional emotions and three-dimensional emotions, and for DEAP, we identified the accuracy of arousal and valence. The results of SEED are shown in Figure 6. Figure 6A shows the recognition accuracy of two-dimensional emotion under the LOSO strategy. The average value of accuracy is 83.87%. Figure 6B shows the recognition accuracy of three-dimensional emotion under the LOSO strategy. The average value of accuracy is 69.76%. Figure 6C shows the recognition accuracy of two-dimensional emotion under the 10-fold CV strategy. Figure 6D shows the recognition accuracy of three-dimensional emotion under the 10-fold CV strategy.

**FIGURE 7:**
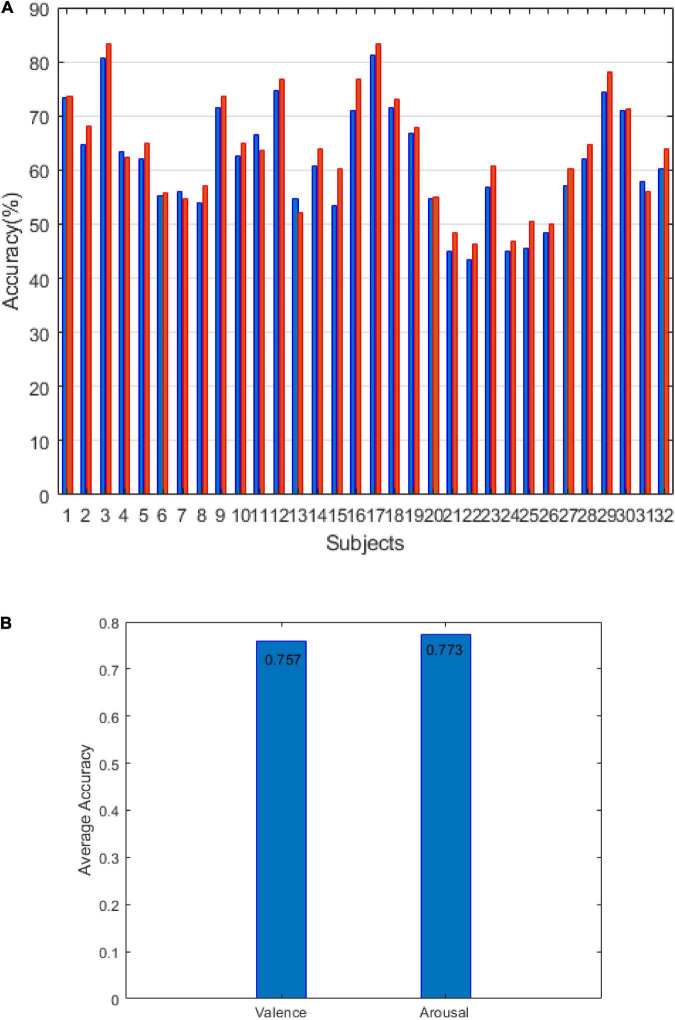
The results of the DEAP dataset: **(A)** the result of LOSO and **(B)** the result of 10-fold CV.

The DEAP results are shown in Figure 7. Figure 7A shows the accuracy of arousal and valence under the LOSO strategy. The average value of arousal was 63.35% and the valence was 61.39%. Figure 7B shows that the accuracy of arousal and valence is under the 10-fold CV strategy. The average value of arousal is 77.3%, and the valence is 75.7%. The results of Figures 6, 7 verify the effectiveness of our method.

(4) Comparison with other methods:

We compared the results obtained by the proposed method with other methods that adopt the same dataset and adopt the same verification strategy. The comparison results are shown in Table 5.

**TABLE 5:** Comparison results.

Dataset	Method	No. of classes	Validation strategy	Accuracy
SEED	ApEn, PerEn, ShEn, etc. +SVM (Li et al., 2018)	2	LOSO	83.33%
	ST-SBSSVM (Yang et al., 2019)	2	LOSO	89%
	Holo-FM/CNN + SVM (Topic and Russo, 2021)	2	10-fold CV	88.45%
	Differential entropy (Zheng et al., 2019)	3	LOSO	60.93
	Our method	2	10-fold CV	89.42%
		3	10-fold CV	81.23%
		2	LOSO	83.87
		3	LOSO	69.76
DEAP	Holo-FM/CNN + SVM (Topic and Russo, 2021)	2	10-fold CV	V:76.61% A:77.72%
	RGB Heat-map/CNN + ELM (S. Siddharth et al., 2019)	2	10-fold CV	V:71.09% A:72.58%
	ApEn, PerEn, ShEn, etc. +SVM (Li et al., 2018)	2	LOSO	59.06
	Our method	2	10-fold CV	V:75.78% A:77.34%
		2	LOSO	V:61.39% A:63.35%

## Discussion

In this study, we proposed the concept of an energy sequence to reduce the influence of noise caused by feature extraction and then construct a dynamic energy feature set including time-domain features, frequency domain features, and non-linear features. The network model adopts fully connected layers and LSTM networks, which are suitable for small datasets. In Figure 1, for the dynamic energy sequence feature set + SVM, the recognition rate is 72.5%. For the traditional feature set + SVM, the recognition rate is 72.2%, which shows that the dynamic energy sequence can preserve the features of the EEG signal and effectively reduce the feature dimension. To further extract effective features, this study comprehensively considers the effectiveness of a single feature for labeling and the information redundancy between multiple features. Through the feature type selection experiment, 17 kinds of feature vectors were selected from the SEED dataset, and 19 kinds of feature vectors were selected from the DEAP dataset. Finally, to verify the feasibility of our method and to facilitate the comparison with advanced methods, this study selects two verification strategies, namely, LOSO and 10-fold CV. For the SEED dataset, with 2-classification with LOSO, in the study by Li et al. (2018), the accuracy is 83.33%; in the study by Yang et al. (2019), it is 89%; and in our method, it is 83.87%. For the 3-classification problem, in the study by Zheng et al. (2019), the accuracy is 60.93%, and in our method, it is 69.76%. For the 2-classification with a 10-fold CV, in the study by Topic and Russo (2021), the accuracy is 88.45%, and in our method, it is 89.42%. For the DEAP dataset, with 2-classification with LOSO, in the study by Li et al. (2018), the average accuracy is 59.06%; for our method V, the average accuracy is 61.39%, and for A, it is 63.35%. With a 10-fold CV, the result of our method (V: 75.78%, A: 77.34%) is better than the result (V: 71.09%, A: 72.58%) in the study by Siddharth et al. (2019) and close to the result (V: 76.61%, A: 77.72%) in the study by Topic and Russo (2021). Therefore, our method is effective. Emotion is a physiological and psychological activity state induced by internal or external stimuli. Although different scalp regions are affected by emotion, when emotion changes, EEG signals will change harmoniously as a whole. Therefore, this study analyses the synchronization of signals in different regions as a whole, which is consistent with the phenomenon that emotional changes will lead to the overall change of EEG signals. The limitation of this study is that taking all channel signals as a whole cannot verify the relationship between different regions and different emotions. In the future study, we will continue to study the noise problem in EEG signal analysis and processing, as well as the relationship between different regions and emotions.

## Conclusion

In this study, the concept of an energy sequence is proposed, and it is verified through theory and experiment that an emotional recognition method based on an energy sequence can reduce the influence of noise and improve accuracy. In addition, due to the small size of the EEG dataset, this study uses the combination of traditional features and a Bi-LSTM deep network. Compared with other traditional methods and CNN deep neural models, this method can achieve higher average accuracy.

## Data Availability Statement

Publicly available datasets were analyzed in this study. This data can be found here: https://bcmi.sjtu.edu.cn/home/seed/downloads.html and http://www.eecs.qmul.ac.uk/mmv/datasets/deap/readme.html.

## Author Contributions

MZ developed the method based on dynamic energy features, performed all the data analysis, and wrote the manuscript. QW advised data analysis and edited the manuscript. JL supervised and led the planning and implementation of research activities and revised the manuscript. All authors contributed to the article and approved the submitted version.

## Conflict of Interest

The authors declare that the research was conducted in the absence of any commercial or financial relationships that could be construed as a potential conflict of interest.

## Publisher’s Note

All claims expressed in this article are solely those of the authors and do not necessarily represent those of their affiliated organizations, or those of the publisher, the editors and the reviewers. Any product that may be evaluated in this article, or claim that may be made by its manufacturer, is not guaranteed or endorsed by the publisher.
